# Beyond Surgical Limits: A Case Report of Follicular Variant of Papillary Thyroid Carcinoma With Persistent Disease Despite Aggressive Surgical Management

**DOI:** 10.7759/cureus.81978

**Published:** 2025-04-09

**Authors:** Saleh Khurshied, Mehrun Nisa, Wafa Abdul Malik, Hassan Mansoor, Rayyan Sabih

**Affiliations:** 1 Otolaryngology-Head and Neck Surgery, Pakistan Institute of Medical Sciences, Islamabad, PAK; 2 Medicine and Surgery, Pakistan Institute of Medical Sciences, Islamabad, PAK; 3 Cornea and Refractive Surgery, Al-Shifa Trust Eye Hospital, Rawalpindi, PAK; 4 Ophthalmology, Al-Shifa Trust Eye Hospital, Rawalpindi, PAK

**Keywords:** clinical symptoms, investigations, thyroid cancer, thyroid cancers surgery, thyroid tissue

## Abstract

The most prevalent type of thyroid gland cancer is called papillary thyroid carcinoma, which typically manifests as a painless thyroid mass in women over 50 years of age. Fine-needle aspiration cytology (FNAC) and ultrasonography (USG) are used to make the diagnosis, and the patient's symptoms and these results determine the course of treatment. We reported a rare case of follicular variant papillary thyroid cancer in a young woman, diagnosed at an unusual age of 22 years, who had three thyroid surgeries intended to completely remove the disease but were unable to do so. The woman was diagnosed with a case of follicular variant of papillary thyroid carcinoma (FVPTC) initially 10 years back, and a hemithyroidectomy was done for diagnostic purposes. Later on, with the diagnosis of FVPTC, a complete thyroidectomy was done at the same time, and the patient recovered uneventfully. For the next 10 years, the patient remained asymptomatic with no complaint and was on oral thyroxine with six-monthly follow-up with thyroid function tests, which were normal. It was 10 years after the complete thyroidectomy that the patient had a neck USG for a nonthyroidal disease, and it was incidentally found that there was a thyroid remnant in the isthmus region even after the complete thyroidectomy, and this silently persisted for more than 10 years; the thyroidal nature of this tissue was confirmed on thyroid scan and computed tomography. The patient had another surgery (3rd surgery) 10 years after the initial two surgeries, but the surgeon could not find any thyroid tissue with the naked eye and shaved off all the tissue in front of the trachea. The histopathology report later showed only muscle and fibroadipose tissue, revealing the inability to surgically remove this resistant carcinoma even after three surgeries. Following that, the case was discussed in a multidisciplinary team meeting, and it was decided that no further intervention was required in this asymptomatic patient, as the patient was biochemically normal and was planned to be closely monitored with routine follow-ups. Thus, there could be a chance of remnant being left behind even after complete thyroidectomy, and the patient may be totally asymptomatic, giving the impression that this tumor was beyond the limits of surgery in our case.

## Introduction

A rare form of cancer, thyroid gland carcinoma, accounts for around 1% of all newly diagnosed malignant conditions. Women are diagnosed with it three times more frequently than men. Fifty is the median age that it affects [1,2]. The four most prevalent types of these carcinomas are follicular, papillary, medullary, and anaplastic. As "well-differentiated" thyroid tumors, papillary carcinomas account for about 80-85% of all thyroid malignancies [3].

Long-term survival rates for papillary thyroid carcinoma (PTC) are over 95% in numerous studies, indicating that most cases have a favorable prognosis. A less favorable disease is associated with the following risk factors: distant metastases, extra thyroid extension, tumor size greater than 4 cm, and patient age of 45 years or older [4]. Over the past few decades, it has become more common [5].

The recognition of follicular variant of papillary thyroid carcinoma (FVPTC) has grown over the past few years. According to reports, it accounts for between 11.8% and 53.3% of all PTC instances [6]. In the United States, the number of thyroid cancer patients doubled between 1997 and 2007 and reached 62450 in 2015. A total of 90% of thyroid malignancies are well-differentiated thyroid tumors, and PTC accounts for 70% of them [7,8].

Even with its high occurrence, FVPTC's clinical behavior and results are still debatable, which makes developing a standard treatment plan difficult. About one-third of all cases are FVPTC, a fairly frequent variation of PTC. It has distinct clinical behavior and resembles a middle-aged entity with characteristics halfway between classical papillary thyroid carcinoma (CPTC) and follicular thyroid carcinoma (FTC). It's interesting to note that these individuals' long-term results are nonetheless great and comparable to CPTC despite the differences in clinical behavior [7].

The most typical way that FVPTC manifests itself is as a painless lump at the thyroid level. As a result of tracheal compression and/or involvement of the recurrent laryngeal nerve, about 20% of patients may report dysphagia or hoarseness. Thyroid function tests will often be normal in these individuals. The diagnosis is made by fine-needle aspiration cytology (FNAC) and ultrasonography (USG). If it is determined that the tumor is unifocal, smaller than 4 cm, and does not exhibit any evidence of lymph node metastases, the last resort is surgery, either a total thyroidectomy or a lobectomy. Unilateral or bilateral neck dissection is advised for patients with advanced primary malignancies, depending on the size of local lymph nodes and the requirement for further staging [1,9]

The American Thyroid Association (ATA) therapy guidelines state that a lobectomy is adequate for tumors under 1 cm, but a total thyroidectomy is recommended for tumors bigger than 4 cm. Thyroid lobectomy is the recommended treatment for low-risk carcinomas that are greater than 1 cm but smaller than 4 cm [10].

We detailed a unique case of FVPTC that presented in a young woman of 22 years of age. She underwent three thyroid surgeries for total removal of this carcinoma but still retained a thyroid remnant in her neck. Thus, making this case rare and unique by making it beyond the limits of surgery, on one hand, and the patient being clinically and biochemically normal, on the other hand.

## Case presentation

**Figure 1:**
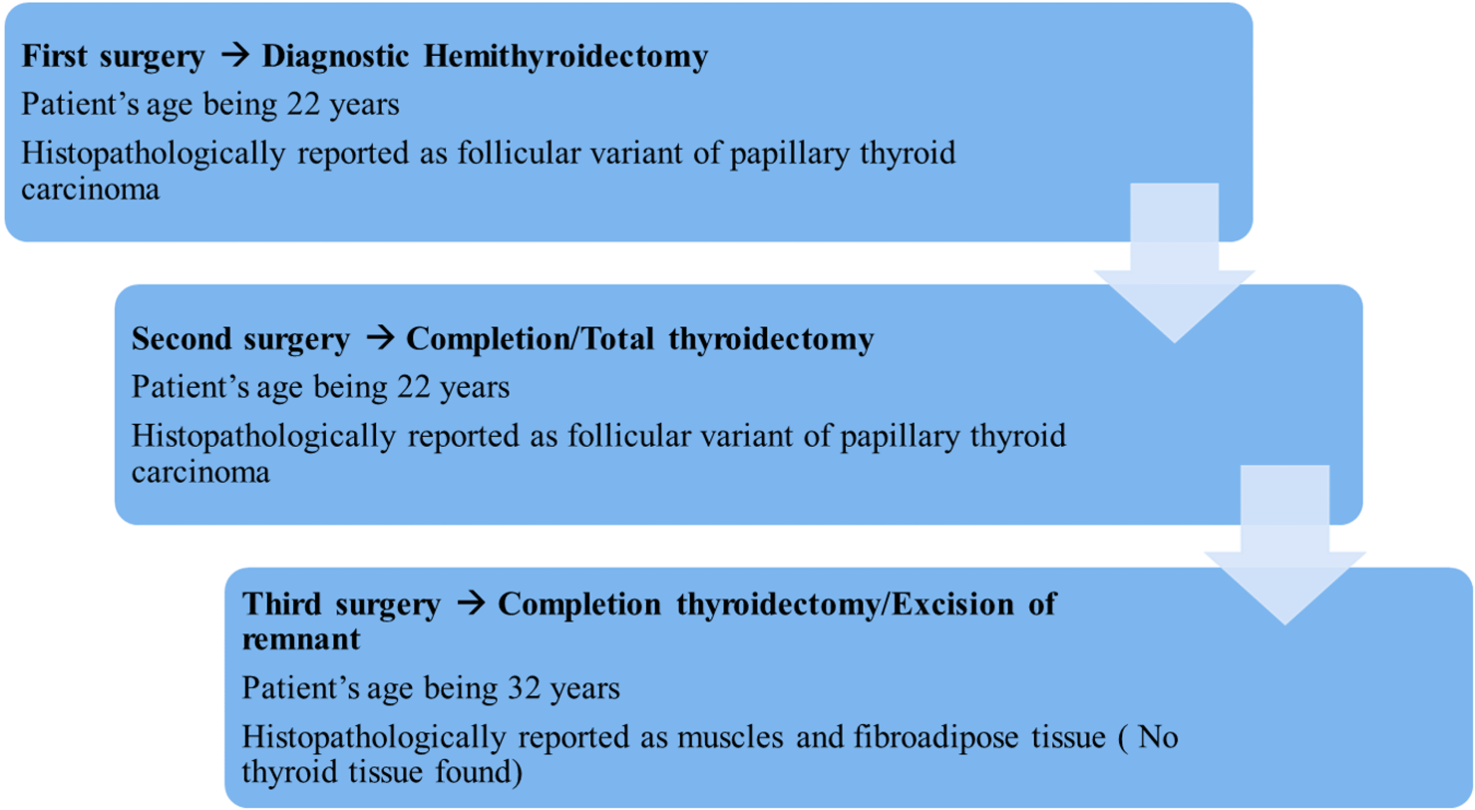
Stepwise surgical intervention done in chronological order

A 22-year-old woman from Punjab province in Pakistan, with no known comorbidities and no family history of thyroid cancer, initially presented 10 years ago in 2014 in the ear, nose, and throat (ENT) outpatient department with a complaint of painless, progressively increasing anterior neck swelling for the last year. Neck examination showed a firm to hard 4 x 4 cm swelling in the center of the neck, mobile on deglutition, nontender with normal color and temperature, noncystic, and irreducible. Thyroid function tests (serum TSH, T4, and T3 levels) were normal; however, FNAC revealed Bethesda III, and USG thyroid performed in October 2014 revealed multinodular goiter TIRAD 3. In February 2015, the patient had a diagnostic hemithyroidectomy, and the histology report showed an FVPTC. In April 2015, the patient underwent a total thyroidectomy due to the diagnosis of FVPTC. The patient recovered completely and well. The patient was started on 50 mcg of thyroxine twice a day orally (BD). Three monthly thyroid function tests were performed, and the results were within the normal range on nearly all follow-ups. A chronologically ordered, step-by-step surgical intervention done on this patient is shown in Figure 1.

**Figure 2:**
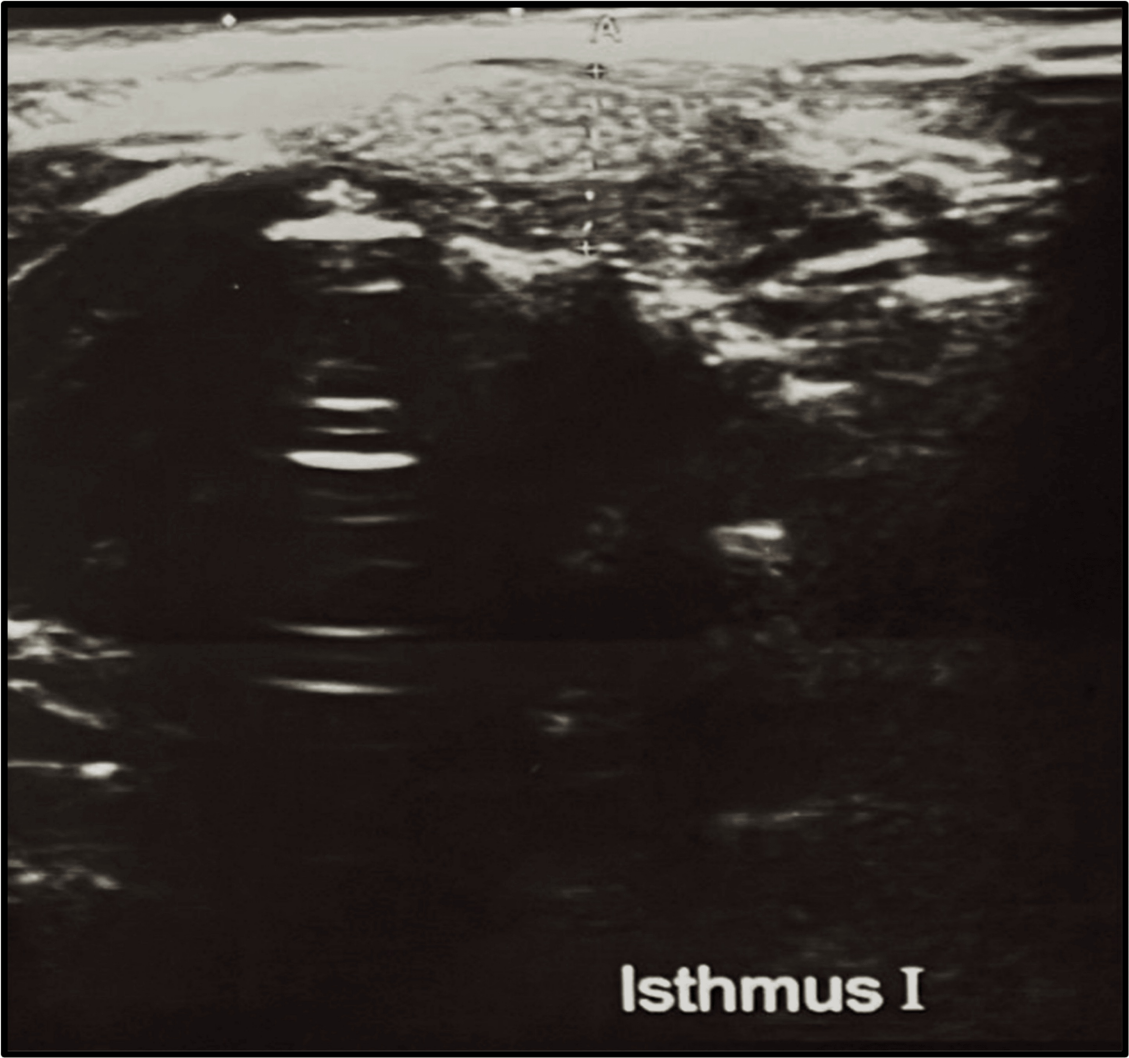
USG thyroid showing thyroid remnant at the level of isthmus (post-total thyroidectomy) USG: ultrasonography

**Figure 3:**
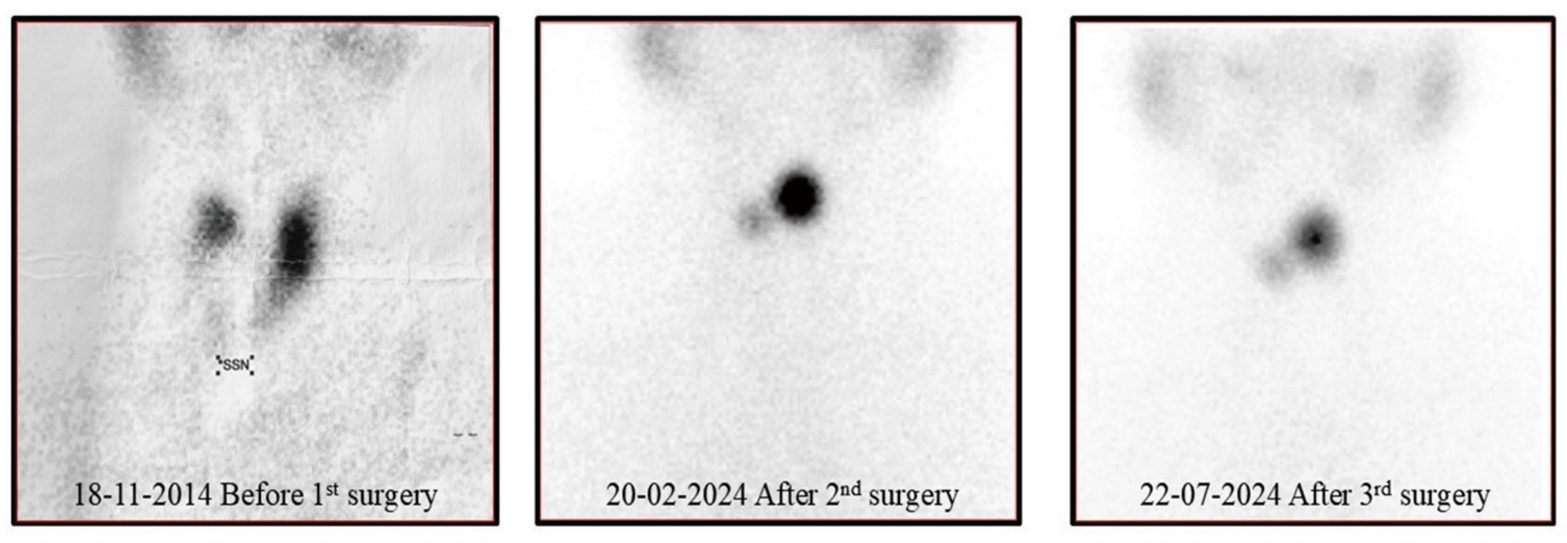
Thyroid scan of patient before and after every surgery, showing constant thyroid remnant

**Figure 4:**
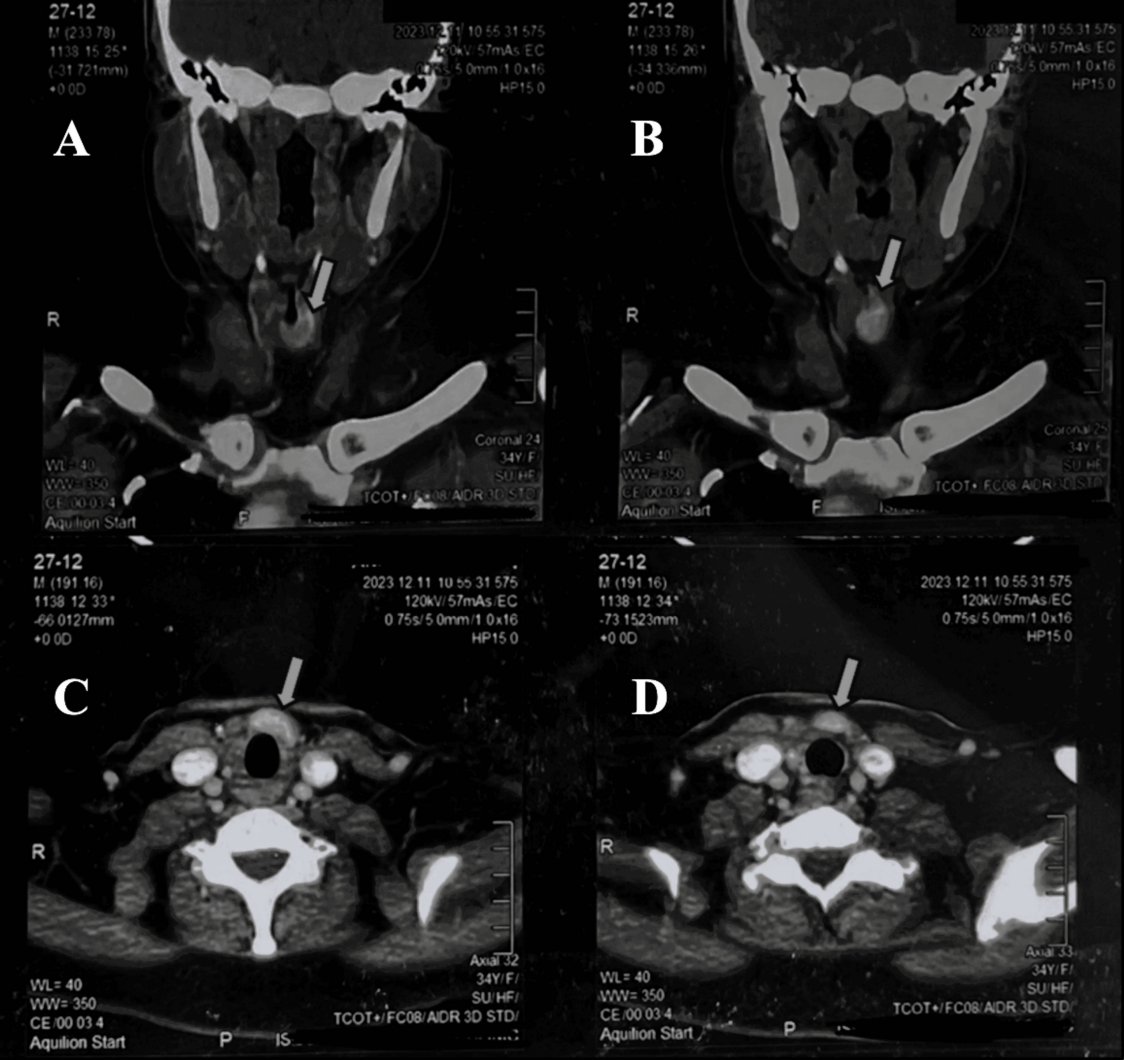
Contrast-enhanced computed tomography neck showing thyroid remnant at the level of isthmus (post-total thyroidectomy) A and B: coronal view showing thyroid remnant as shown by the arrow; C and D: axial view showing thyroid remnant as shown by arrow

The patient was doing well until November 2023, at which point she began experiencing intermittent dizziness, shortness of breath when walking, and generalized weakness. The patient saw a general practitioner, who ordered a full blood count since the patient appeared pale and had a USG thyroid scan, even though the patient did not exhibit any other thyroid disease symptoms, such as neck pain or swelling. The result showed that the patient’s hemoglobin was 9.4 mg/dl, and coincidentally, USG revealed a thyroid remnant in the isthmus measuring 1.7 x 0.7 cm, as shown in Figure 2. The patient had a thyroid scan that showed remnant tissue in the isthmus, as shown in Figure 3. Contrast-enhanced computed tomography (CECT) of the head and neck showed a thyroid remnant in the region of the isthmus measuring 0.86 x 1.44 cm, as shown in Figure 4. The antithyroid antibodies and tumor markers for thyroid cancer came out to be negative.

**Figure 5:**
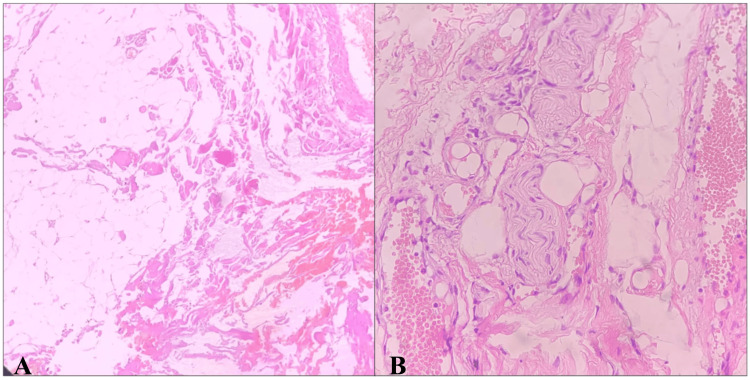
Histopathology picture of completion thyroidectomy specimen without any evidence of thyroid tissue A: photomicrograph showing fibroadipose tissue with entrapped skeletal muscle bundles; B: photomicrograph showing nerve bundle with fibroadipose tissue and dilated vessels

The patient underwent a complete thyroidectomy with bilateral level 1 neck dissection in June 2024. The surgeons' perioperative examinations showed that there was no obvious thyroid remnant visible at the site shown in radiological investigations, and the surgeon removed all the visible tissue in front of the trachea and from the potential remnant location. The histopathology report, as shown in Figure 5, showed muscle fiber and fibroadipose/cartilaginous tissue and no histological evidence of any thyroid tissue; the lymph nodes were also free of tumors.

The case was discussed in a multidisciplinary team meeting with representatives from the fields of oncology, nuclear medicine, ENT-head and neck surgery, endocrinology, and internal medicine. Given that the patient was symptomless and had normal thyroid function test results, negative thyroid antibodies and tumor markers, and no nodal or lymphovascular invasion, the patient was put on regular follow-up with no further surgical intervention or radioactive iodine ablation to be done for now.

## Discussion

This case report details a distinct manifestation of thyroid follicular variant papillary cancer because of the patient's unusual demographics. We presented an unusual case of FVPTC in a 22-year-old young female patient, where this carcinoma silently survived for over 10 years even after having three thyroid surgeries with the aim of totally eradicating the disease but failing to do so. This symptomless patient was then kept on regular follow-ups with close monitoring. PTC commonly affects women (between 2:1 and 4:1), and the median age at which a patient usually presents is 50 years [1,2]. The patient we described was a 22-year-old woman who was otherwise healthy. Furthermore, in PTC, the tumor size is usually around 1 cm to 3 cm, but in our case, it was exceptionally larger, measuring approximately 5 x 3 x 2 cm [11].

For patients with FVPTC with tumors measuring more than 1.5 centimeters, lobectomy or total thyroidectomy are the two main possible surgical options [12]; some doctors advise a complete thyroidectomy, and so that was the surgical plan in our case, where we aimed for total thyroidectomy. Additionally, recommendations state that a lobectomy suffices for the encapsulated, noninvasive FVPTC [13].

To minimize the amount of residual thyroid tissue, total/near thyroidectomy is advised by the 2015 ATA guidelines and in certain papers [14]. This procedure can increase the likelihood of a successful total removal and reduce the rates of recurrence, distant metastasis, and mortality. Therefore, we kept the patient on regular follow-ups instead of having a thyroid remnant in the end. Holsinger et al. [15] in their research concluded that thyroid remnants are nearly always present, despite surgeons' radical intentions, and the removal of all thyroid tissue via complete thyroidectomy is still a "myth" and so was in our case.

According to Pan et al.'s study [16], only 2.2% of patients had a true total thyroidectomy, meaning that no remnant was discovered after the procedure. The rest of the patients, however, had some thyroid remnant that was visible using any imaging modality. In addition, a substantial thyroid remnant was seen in 14.7% of patients, which is what happened in our instance. In their study of all thyroidectomy patients, Salvatori et al. [17] discovered that 93.1% of patients had thyroid remnants. However, even after three thyroid surgeries, our patient experienced no surgical issues.

According to Zidan et al. [18], there is disagreement over the ideal surgical extent. According to them, the proportion of patients who had a total thyroidectomy and a subtotal thyroidectomy was comparable in both PTC and FVPTC, and the survival rates of the thyroidectomy and subtotal thyroidectomy groups were identical.

## Conclusions

This case report illustrates an unusual presentation and resistance to complete removal of FVPTC even after multiple surgeries. Thus, there could be a chance of remnant being left behind even after complete thyroidectomy, and the patient may be totally symptomless, giving the impression that this tumor was beyond the limits of surgery in our case. Also, the literature review gave the impression that it is not uncommon to have a thyroid remnant after total thyroidectomy for any reason; most of the time, it's an incidental finding.
